# Self-Assembled pH-Responsive Metal-Organic Frameworks for Enhancing the Encapsulation and Anti-Oxidation and Melanogenesis Inhibition Activities of Glabridin

**DOI:** 10.3390/molecules27123908

**Published:** 2022-06-18

**Authors:** Liang Chen, Zexun Liu, Xinying Zhao, Linying Liu, Xiulan Xin, Hao Liang

**Affiliations:** 1 College of Bioengineering, Beijing Polytechnic, Beijing 100176, China; zhaoxinying@bpi.edu.cn (X.Z.); liulinying@bpi.edu.cn (L.L.); xinxiulan@bpi.edu.cn (X.X.); 2 State Key Laboratory of Chemical Resource Engineering, Beijing University of Chemical Technology, Beijing 100029, China; liuzexun@mail.buct.edu.cn; 3 Qinhuangdao Bohai Biological Research Institute, Beijing University of Chemical Technology, Qinhuangdao 066000, China

**Keywords:** self-assembly, pH-controlled release, metal organic frameworks (MOFs), glabridin, encapsulation, anti-oxidation, melanogenesis inhibition

## Abstract

Metal organic frameworks (MOFs) are formed by self-assembly of metal ions and organic ligands. A special type of MOF called ZIF-8, which is formed by self-assembly of zinc ions and 2-methylimidazole, shows excellent stability in aqueous solutions and disintegrates under acidic conditions. These properties make ZIF-8 a suitable carrier material for pH-stimulated drug delivery systems. Glabridin is an isoflavane compound that is widely present in the roots of *licorice*. Because of its outstanding skin whitening properties, glabridin is widely used as a whitener in the cosmetics industry. In this study, ZIF-8 was employed to encapsulate glabridin. Glabridin-loaded ZIF-8 was successfully prepared with a drug encapsulation efficiency of 98.67%. The prepared sample showed a fusiform or cruciate flower-like structure, and its size was about 3 μm. ZIF-8 enabled pH-controlled release of glabridin. Moreover, ZIF-8 encapsulation significantly enhanced the intracellular anti-oxidant activity and melanogenesis inhibitory activity of glabridin. This study provides a new approach that shows great potential to improve the biological application of glabridin.

## 1. Introduction

**Figure 1:**
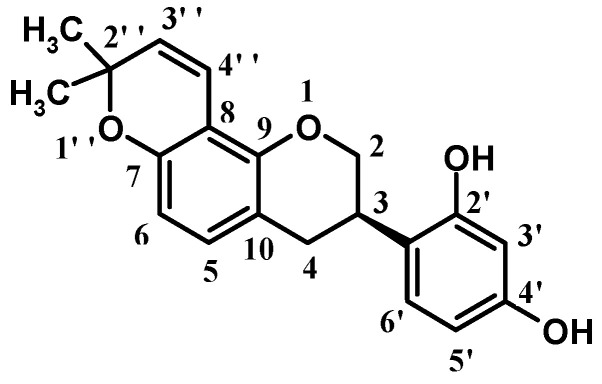
Structure of glabridin. Numbers on the Structure of glabridin are used to distinguish the same functional groups at different positions.

Glabridin (Figure 1) is an isoflavane compound that is widely present in the roots of *licorice*. It is a non-polar molecule that poorly dissolves in water but easily dissolves in organic solvents such as ethanol, propylene glycol, and butylene glycol. Many clinical studies have shown that glabridin has a wide range of biological activities [1,2]. For example, glabridin has been shown to have anti-oxidation, anti-inflammation, cardiovascular protection, neuro-protection, energy expenditure, metabolism regulation, and hypoglycemic effects [3,4,5,6,7,8,9]. Moreover, recent studies have reported that glabridin has anti-cancer activity [10,11].

In particular, glabridin has outstanding skin whitening properties [12]. Inhibitors of tyrosinase, a key rate-limiting enzyme in the melanin biosynthesis pathway [13,14], have a significant therapeutic effect on skin diseases caused by abnormal pigmentation disorders; thus, they are added to cosmetics as whitening ingredients [15]. Yokota et al., reported that in B16 mouse melanoma cells, the activities of tyrosinase T1 and T3 were inhibited after treatment with glabridin [16]. When *guinea* pigs were topically treated with 0.5% glabridin, the degree of erythema and pigmentation caused by UV_B_ were significantly reduced [17,18]. Structure-activity relationship studies indicated that the 2′- and 4′-hydroxy groups of glabridin are the active sites responsible for its inhibitory effect on tyrosinase [16].

In recent years, many articles and patents have been devoted to the application of glabridin in whitening [19,20]. However, glabridin also possesses many disadvantages, such as poor water solubility, low chemical stability, fast metabolism, and a short half-life; it is easily decomposed when exposed to light and heat, making it difficult to be absorbed and utilized by organisms. There is an urgent need to develop a safer and more efficient drug delivery system for glabridin in order to solve the problems of low drug efficacy and non-targeted release during the process of drug delivery.

One possible approach is to deliver glabridin using metal organic frameworks (MOFs), which are formed by self-assembly of metal ions and organic ligands. Because they possess properties such as high porosity, a large specific surface area, and an adjustable pore size, MOFs have been more and more frequently used as drug delivery system carriers in biological research. They can deliver large amounts of drugs to target cells. However, most MOFs are not stable and they easily disintegrate in aqueous solutions. Therefore, there are many challenges when seeking suitable MOFs for drug delivery vehicles [21,22,23]. Among the MOFs, ZIF-8, which is formed by self-assembly of zinc ions (Zn^2+^) and 2-methylimidazole (2-MIM), is special. In addition to possessing the advantages of other MOFs, ZIF-8 shows high stability in aqueous solutions due to the strong interaction between Zn^2+^ and 2-MIM. But it is prone to disintegration under acidic conditions, which can be used as carrier material for pH-stimulated drug delivery systems [24]. For instance, Zhang et al., co-loaded the anti-cancer drugs doxorubicin and verapamil into ZIF-8 and studied the pH-responsive drug release behavior of this system [25]. In a study by Yang et al., ZIF-8 loaded with doxorubicin and camptothecin, and the pH/near-infrared dual-stimulus-responsive drug release behavior in liver cancer cells was evaluated [26]. Chen et al., loaded the autophagy inhibitor 3-methyladenine into ZIF-8 and studied the relationship between anti-tumor efficacy and inhibition of autophagy in Hela cells [27].

The methods for synthesis of ZIF-8 can be roughly divided into the following two categories. The first method is the addition prepared ZIF-8 to drug-containing solvent to achieve the coating of drug molecules through ZIF-8 adsorption [28]. However, this process is only suitable for small-molecule drugs with a particle size smaller than the pore size of ZIF-8, and drugs with larger particles cannot be effectively encapsulated. In addition, drugs loaded through adsorption easily leak during delivery, and burst release behavior easily occurs as well. The second method is one-step in-situ encapsulation; that is, the drug molecules are loaded into ZIF-8 during self-assembly. In this way, coating problems due to drug molecule size can be avoided, and drug leakage and release are not likely to occur. But this scheme is limited to those drug molecules, such as doxorubicin, camptothecin, and caffeine, that have specific functional groups such as –NH_3_, –COOH, and –SO_3_H [29,30,31]. To overcome these limitations, we tried to use a seed growth method to achieve encapsulation of glabridin by ZIF-8, which has been successfully applied for encapsulation of hydrophobicity compounds [32].

In this study, we successfully generated glabridin-loaded ZIF-8 (Gla-ZIF-8) and characterized its encapsulation rate, morphology, and performance. The structural characteristics and biological activities of the drug molecules before and after encapsulation were also studied. Furthermore, the applicability of the ZIF-8 drug loading system, which employs crystal seeded growth, to hydrophobic drugs was investigated.

## 2. Results and Discussion

### 2.1. Preparation of Gla-ZIF-8

A simple anti-solvent precipitation method was used to prepare Gla-ZIF-8, which contains the highly hydrophobic compound glabridin. First of all, glabridin dissolved in ethanol was gradually added into a Zn^2+^ aqueous solution. Microcrystal particles of glabridin were formed. Then, 2-MIM was added to the above mixture, and it coordinated with Zn^2+^ to form ZIF-8. During this stage, ZIF-8 grew on the surface of the microcrystal particles and at the same time encapsulated glabridin. After a period of continuous growth, aggregation, and sedimentation, the sample was centrifuged, and a white precipitate was obtained. The successful preparation of Gla-ZIF-8 shows that the seeded-grown ZIF-8 loading process is suitable for the construction of delivery systems for hydrophobic drugs in the aqueous phase. The process is not only simple and effective, but also green and safe.

### 2.2. Encapsulation Efficiency of Gla-ZIF-8

The DLE of Gla-ZIF-8, calculated using Equation (1), was 98.67 ± 0.43%. This high DLE, combined with the similarly high DLE reported previously for curcumin (CCM)-ZIF-8 (98.21 ± 1.02%) [33], demonstrates that the seeded-grown ZIF-8 loading process can achieve a very high drug encapsulation efficiency [32].

There are two reported ZIF-8 drug loading modes [30,31]. One mode is to load drugs through adsorption. The drug encapsulation efficiency depends on the size and quantity of the pores of the carrier. This mode will lead to low encapsulation efficiency. In addition, the drug is easily adsorbed on the surface of the carrier, which results in leakage in the process of drug delivery. Another mode is to load drugs in the self-assembly process of ZIF-8. There must be a strong interaction between drug molecule and carrier. This mode depends on whether the drug molecule itself has specific functional groups such as -NH_3_, –COOH, and –SO_3_H. The preparation method of seed growth developed in this study avoids these problems. The ZIF-8 drug-loading system, which has high encapsulation efficiency, could be used for all hydrophobic drugs.

### 2.3. Characterization of Gla-ZIF-8

**Figure 2:**
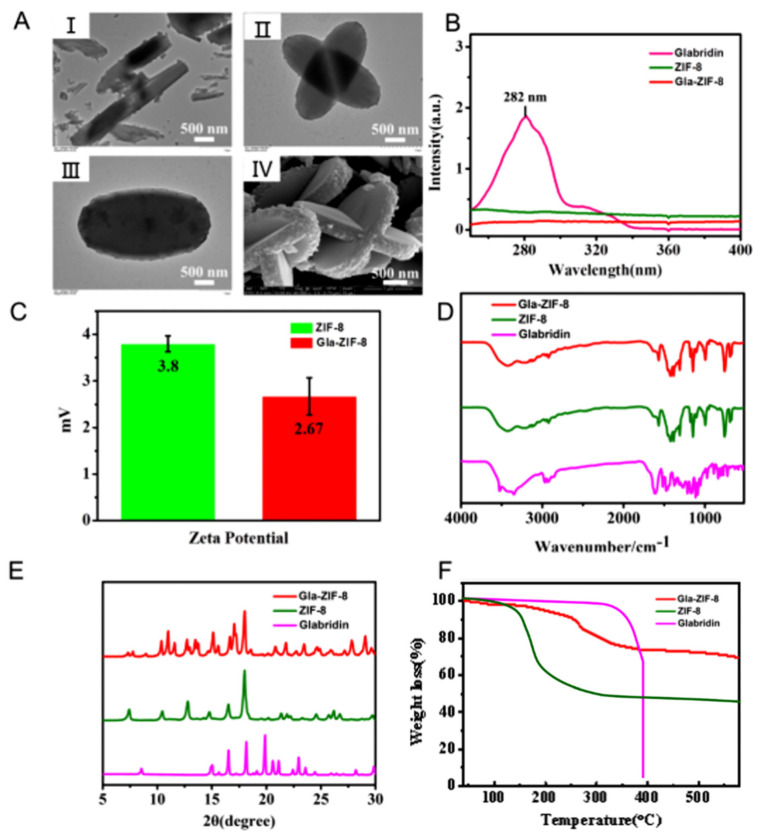
Physicochemical properties of Gla-ZIF-8. (**A**) TEM images of microcrystals of glabridin (I) and Gla-ZIF-8 (II, III) and an SEM image of Gla-ZIF-8 (IV). (**B**) UV-Vis spectra of glabridin, ZIF-8, and Gla-ZIF-8. (**C**) Zeta potentials of ZIF-8 and Gla-ZIF-8. (**D**) FTIR spectra of glabridin, ZIF-8, and Gla-ZIF-8. (**E**) XRD patterns of glabridin, ZIF-8, and Gla-ZIF-8. (**F**) TGA curves of glabridin, ZIF-8 and Gla-ZIF-8.

Transmission electron microscopy (TEM) and scanning electron microscopy (SEM) were used to characterize the formation and morphology of Gla-ZIF-8. Morphological analysis revealed that glabridin microcrystals that formed in aqueous solution appeared as prismatic shapes (Figure 2A(I)). Subsequently, ZIF-8 adhered to the surface of the crystal and grew and aggregated, coating the drug and thereby forming Gla-ZIF-8. From the TEM images (Figure 2A(II,III)) and the SEM image (Figure 2A(IV)) of Gla-ZIF-8, we can see that it has a stick-like fusiform or a cruciate flower-like structure. The size of Gla-ZIF-8 was about 3 μm.

The UV-Vis spectra of glabridin, ZIF-8, and Gla-ZIF-8 are shown in Figure 2B. There was a distinguishing maximum absorption peak at 282 nm in the UV-Vis spectrum of glabridin. However, there was no absorbance at the same wavelength in the UV-Vis spectra of ZIF-8 and Gla-ZIF-8. These results indicated that the characteristic peaks of glabridin were masked and that glabridin was encapsulated successfully into the ZIF-8 frame. In addition, the zeta potential of Gla-ZIF-8 (+2.67 mV) was similar to that of ZIF-8 (+3.8 mV) (Figure 2C), and both show positive electricity, confirming that glabridin was loaded into ZIF-8 tightly. The FT-IR spectra of glabridin, ZIF-8, and Gla-ZIF-8 are shown in Figure 2D. The FT-IR spectrum of glabridin contained specific peaks at 3340 cm^−1^ (O–H stretching vibration), 1520 cm^−1^ and 1480 cm^−1^ (C–C stretching vibration in aromatic ring), and 1270 cm^−1^ (stretching of C–O–C). However, the characteristic peaks of ZIF-8 appeared at 3177 cm^−1^ (aromatic C–H stretching) and 1566 cm^−1^ (C–N stretching of imidazole). The peaks of Gla-ZIF-8 at 3220 cm^−1^ and 1568 cm^−1^ corresponded to the aromatic C–H stretching and C–N stretching of imidazole, respectively, which are similar to the characteristic peaks of ZIF-8. However, the characteristic peaks of glabridin were not present in the Gla-ZIF-8 spectrum, indicating that glabridin was effectively encapsulated by ZIF-8. The correct formation of Gla-ZIF-8 was further supported by its XRD patterns (Figure 2E). The characteristic peaks of ZIF-8 were at 2θ values of 10.54, 12.80, and 18.00. Glabridin displayed several sharp peaks at diffraction angles of 15.02, 16.54, 18.16, 19.88, 20.06, and 21.6. The XRD pattern of Gla-ZIF-8 had the characteristic peaks of ZIF-8 (10.38 and 12.72), while the glabridin peak at 19.88 disappeared, indicating that glabridin was successfully encapsulated by ZIF-8. According to our previous study, CCM-ZIF-8 has XRD peaks similar to those of Gla-ZIF-8 (at 10.38, 11.00, 11.58, 12.72, 13.48, 15.12, 16.66, 17.06, and 18.00), indicating that drug-loaded ZIF-8 forms unique crystals and has a special structure [32].

The TGA curves of glabridin, ZIF-8 and Gla-ZIF-8 are shown in Figure 2F. In the temperature range of 40~350 °C, the weight loss of glabridin was only 6.54%, while the weight loss of Gla-ZIF-8 was 24.31%. Accordingly, it can be speculated that part of the loss in Gla-ZIF-8 is derived from ZIF-8. When the temperature was increased from 350 °C to 390 °C, free glabridin was degraded rapidly, and its weight loss was as high as 88.69% in 40 min. In contrast, when the temperature rose to 580 °C, 69.30% of Gla-ZIF-8 still existed stably. The results indicated that the protective encapsulation by ZIF-8 enhanced the thermal stability of glabridin and effectively prevented its rapid decomposition at high temperature.

### 2.4. In Vitro pH-Responsive Release of Glabridin from Gla-ZIF-8

**Figure 3:**
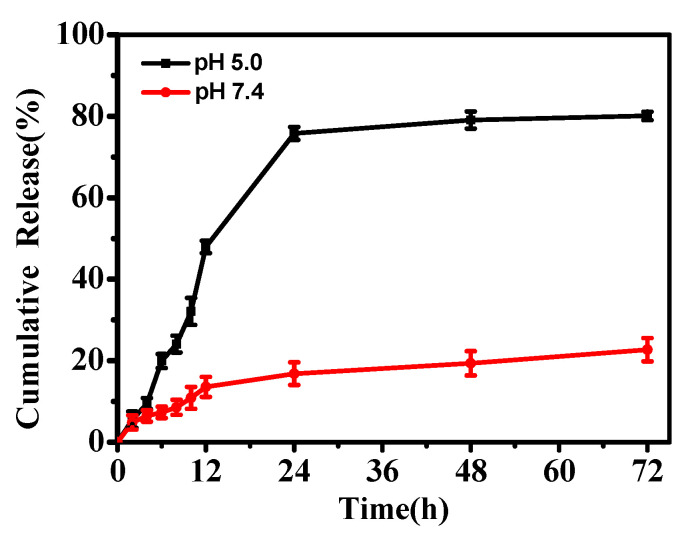
The in vitro release curves of glabridin from Gla-ZIF-8 in PBS (pH 7.4 and 5.0).

The pH-responsive release of glabridin from Gla-ZIF-8 was investigated in an in vitro drug-release experiment, which was carried out in PBS solution under different pH conditions (pH 5.0 to represent the acidic conditions in tumor cells and pH 7.4 to represent the physiological pH). To improve the solubility of glabridin, 10% ethanol was added to the PBS solution. As Figure 3 shows, the release rate for Gla-ZIF-8 at pH 5.0 was sustained and efficient; the cumulative release of Gla-ZIF-8 reached 80.08% after 72 h in PBS at pH 5.0. By contrast, the release rate for Gla-ZIF-8 at pH 7.4 was very slow; only 20.82% of glabridin was released after 72 h. The pH-controllable drug release properties of Gla-ZIF-8 could be exploited for the development of delivery systems for irritating drugs.

### 2.5. Tyrosinase Inhibitory Activity

**Figure 4:**
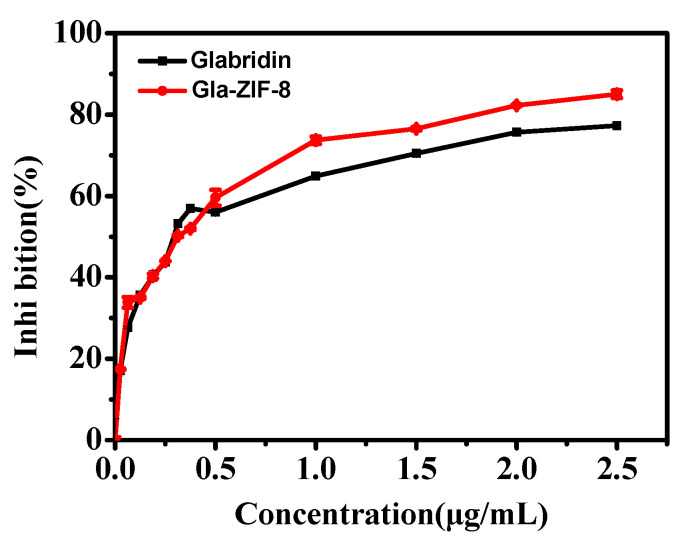
Tyrosinase inhibitory activities of free glabridin and Gla-ZIF-8.

Regulating the catalysis of L-DOPA by tyrosinase is the key to controlling the production of melanin, and glabridin is known to inhibit the activity of tyrosinase. The results of tyrosinase inhibitory activity assays of glabridin and Gla-ZIF-8 are shown in Figure 4. For the evaluated range, with increasing concentrations of glabridin, the percentage of tyrosinase inhibition activity of both free drug and Gla-ZIF-8 increased. Specifically, when the concentration of glabridin was lower than 0.5 μg/mL, there was no significant difference in the inhibition percentage between the two samples. When the concentration of glabridin was 1~2.5 μg/mL, the inhibition percentage of Gla-ZIF-8 was higher than that of the free drug. This may be due to the conformation of the inclusion complexes, which increased the dispersibility of Gla-ZIF-8 in the PBS solution. Conversely, free glabridin had poor water solubility and a low degree of dispersion in the aqueous phase, resulting in a poor inhibitory effect on melanin production.

### 2.6. In Vitro Anti-Oxidant Activity of Gla-ZIF-8

**Figure 5:**
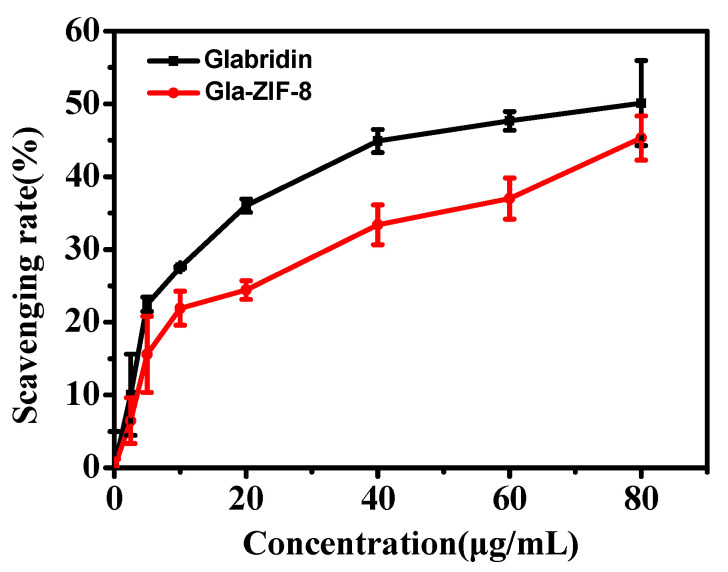
DPPH radical-scavenging capacities of free glabridin and Gla-ZIF-8.

To evaluate the anti-oxidant activities of free glabridin and Gla-ZIF-8, a DPPH radical-scavenging capacity experiment was carried out. As shown in Figure 5, the scavenging capacities of glabridin and Gla-ZIF-8 increased with increasing concentration within the evaluated range. When the concentration of glabridin was 80 µg/mL, there was no significant difference between the scavenging capacities of glabridin and Gla-ZIF-8. Overall, the difference in DPPH radical-scavenging capacity between free glabridin and Gla-ZIF-8 was no significant.

### 2.7. Cellular Anti-Oxidant Activity

The CAAs of glabridin, ZIF-8, and GLA-ZIF-8 in MGC-803 cells were evaluated [33,34,35]. DCFH-DA is a cell-permeable probe used to detect intracellular reactive oxygen species (ROS). ROS are internalized by cells and converted to DCFH via intracellular esterases. Furthermore, non-fluorescent DCFH is oxidized by ABAP into 7′-Dichlorofluorescein (DCF), which has fluorescent properties, and the fluorescence intensity of DCF reflects the degree of oxidative damage caused by radicals. Conversely, after the addition of anti-oxidants, the anti-oxidant capacity of the drug is reflected according to the degree of decrease in the DCF fluorescence intensity. In this study, MGC-803 cells were chosen as an oxidative cell model to assess the CAAs (calculated according to Equation (4)) of free glabridin, ZIF-8, and Gla-ZIF-8.

**Figure 6:**
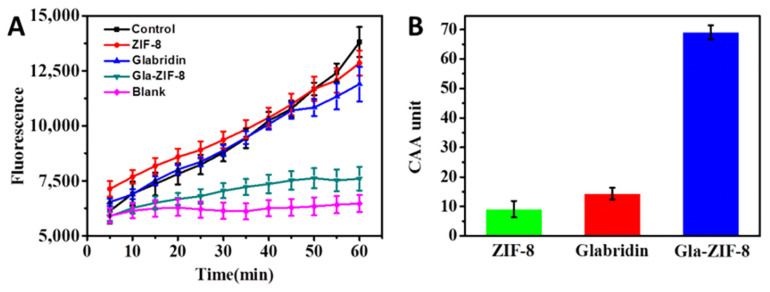
Kinetics curve of DCF fluorescence (**A**) and CAA values (**B**) of free glabridin, ZIF-8, and Gla-ZIF-8.

The DCF fluorescence kinetics curves of glabridin, ZIF-8, and Gla-ZIF-8 are shown in Figure 6A. Only DCFH-DA and ABAP were added to the control group, and only ABAP was added to the blank group. The gradual increase in fluorescence intensity indicated that ABAP continuously oxidized DCFH to DCF. As an anti-oxidant, glabridin could block this process. In particular, the smoother the trend of the DCF fluorescence kinetic curve, the higher the anti-oxidant activity of the exogenous additives. Compared with free glabridin and ZIF-8, Gla-ZIF-8 exhibited stronger anti-oxidant capacity; the CAA value of Gla-ZIF-8 (69.08) was much higher than that of free glabridin (14.35) and ZIF-8 (9.12) (Figure 6B). The difference in CAA between Gla-ZIF-8 and glabridin was significant. This indicates that the CAA of glabridin was enhanced by encapsulation in ZIF-8 and the transcellular transport of glabridin by ZIF-8. This enhancement might be due to the electrostatic interaction between the positive potential on the surface of Gla-ZIF-8 and the negative potential on the cell membrane surface, which promoted the absorption of Gla-ZIF-8 by cells.

## 3. Materials and Methods

### 3.1. Materials

Glabridin (98% purity) was obtained from Jiamu Biotechnology (Hunan, China); 2-methylimidazole (2-MIM), zinc nitrate hexahydrate (Zn(NO_3_)_2_·6H_2_O), and 2,2-diphenyl-1-picrylhydrazyl (DPPH) were purchased from Energy Chemical (Shanghai, China); 2,2-Azobis (2-amidinopropane) dihydrochloride (ABAP) and 2,7-dichlorodi-hydrofluorescein diacetate (DCFH-DA) were purchased from Sigma-Aldrich (St. Louis, MO, USA); tyrosinase (25 KU, from mushroom) and 3,4-dihydroxy-l-phenylalanine (L-DOPA) were purchased from Zhiji BioChemical (Shanghai, China); Cell counting kit-8 (CCK-8) was purchased from MP Biomedicals (Santa Ana, CA, USA); Dulbecco’s Modified Eagle Medium (DMEM) culture media and fetal bovine serum (FBS) were purchased from Gibco (Grand Island, NY, USA); 0.25% pancreatin (containing 0.53 mM EDTA·4Na), phosphate-buffered saline (PBS, 0.01 M, pH 7.4), penicillin (10,000 U/mL), and streptomycin (10,000 U/mL) were purchased from Hyclone (Logan, UT, USA). All other analytical grade chemicals were purchased from Beijing Chemical Works (Beijing, China).

The human gastric cancer cell line MGC-803 was obtained from the Cell Resource Center, Peking Union Medical College (Beijing, China), and cultured in DMEM culture media, which was supplemented with 10% FBS and 1% penicillin-streptomycin, in a 5% CO_2_ atmosphere under a temperature of 37 °C. The time interval for changing the medium was 24 h. The adherent cells were digested with 0.25% trypsin, and the cells were collected by centrifugation at 1000 rpm and then passaged or used.

### 3.2. Analysis by Reverse-Phase High-Performance Liquid Chromatography

Reverse-phase high performance liquid chromatography (RP-HPLC) (LC-20A, Shimadzu, Kyoto, Japan) was carried out on a Venusil XBP C18 analytical column (4.6 mm × 250 mm, 5 μm), which was placed in a column oven with the temperature set at 30 °C, and the mobile phase was acetonitrile—2% acetic acid in water (50:50, *v*/*v*). The UV detection wavelength was set at 282 nm and the flow rate was 1.0 mL/min, with an injection volume of 20 μL. A commercially available standard of glabridin was used to create a calibration curve. Standard solutions of glabridin were prepared with ethanol at concentrations of 20, 40, 80, 100, 160, and 200 μg/mL, and then RP-HPLC was used to determine the concentration of glabridin. The glabridin standard curve was drawn, which yielded the following calibration equation:

Y = 22313X + 44896 (R^2^ = 0.9996)

### 3.3. Preparation of Gla-ZIF-8

The anti-solvent precipitation method was used to prepare Gla-ZIF-8 according to a previous report [32]. In brief, under vigorous stirring, 10 mL of glabridin-ethanol solution (2 mg/mL) was added into 50 mL of Zn(NO_3_)_2_·6H_2_O aqueous solution (30 mg/mL). Then 50 mL of 2-MIM aqueous solution (66 mg/mL) was added. The whole mixture was reacted for 30 min at room temperature. Next, the reaction mixture was centrifuged, and the white precipitate (Gla-ZIF-8) was thoroughly washed twice with deionized water to remove the residue on the surface of Gla-ZIF-8. Powdered Gla-ZIF-8 was finally obtained by freeze-drying under vacuum.

To calculate the amount of Gla-ZIF-8 encapsulated, 10 mg of Gla-ZIF-8 powder was first dissolved in 200 μL HCl (1 M) and then diluted with ethanol to a volume of 2 mL. The supernatant was collected to determine the drug-loading efficiency (DLE) of glabridin (Equation (1)):

(1) $$\mathrm{DLE}=\frac{\mathrm{amount}\;\mathrm{of}\;\mathrm{glabridin}\;\mathrm{retained}\;\mathrm{in}\;\mathrm{Gla-ZIF-8}}{\;\mathrm{total}\;\mathrm{amount}\;\mathrm{of}\;\mathrm{of}\;\mathrm{glabridin}\;\mathrm{added}}\times 100\%$$

### 3.4. Characterization of Gla-ZIF-8

The UV-Vis absorption spectra of a glabridin-methanol solution, ZIF-8 aqueous solution, and Gla-ZIF-8 aqueous solution were recorded using a UV-2450 spectrophotometer (Shimadzu, Kyoto, Japan). A small amount of Gla-ZIF-8 aqueous solution was added dropwise to a copper mesh/silicon wafer. After drying, the morphology and size of Gla-ZIF-8 were recorded using a Hitachi HT7700 transmission electron microscope and a Hitachi S-4700 scanning electron microscope (Hitachi, Tokyo, Japan), respectively. The zeta potentials of the ZIF-8 and Gla-ZIF-8 aqueous solutions were recorded by a nanoparticle size potential analyzer (Nano-ZS 2000, Malvern, UK). Fourier-transform infrared (FT-IR) spectra of glabridin, ZIF-8, and Gla-ZIF-8 were recorded on a Nicolet 6700 FT-IR spectrometer (Thermo Fisher Scientific, Waltham, MA, USA) in the range of 4000~400 cm^−1^. The appropriate amounts of dry powder of glabridin, ZIF-8, and Gla-ZIF-8 were loaded on the sample stage, and the differences in crystal structures were analyzed by comparing X-ray diffraction (XRD) patterns obtained using an X-ray diffractometer (BRUKER-AXS, Bruker, Germany). The appropriate amounts of dry powder of glabridin and Gla-ZIF-8 were placed in a sample crucible, and the powders were analyzed using thermogravimetric analysis (TGA) (DSC1, Mettler, Switzerland) in the range of 25~1000 °C.

### 3.5. In Vitro Drug Release Behavior of Gla-ZIF-8

The in vitro drug release behavior of Gla-ZIF-8 was evaluated according to a previous publication [25]. In brief, 10 mg of Gla-ZIF-8 powder was dispersed into 50 mL of a PBS solution (0.02 M, 10% ethanol). Considering the poor solubility of glabridin in water, Tween-80 (1.0 wt%) was added to the PBS solution to improve its stability. The whole mixture was kept at 37 °C with continuous shaking, then centrifuged. A specific volume of supernatant was collected at predetermined times for HPLC analysis, and an equal volume of fresh solution was added. The cumulative release of glabridin (expressed as the relative percentage released) was calculated as a function of incubation time. All experiments were carried out in triplicate.

### 3.6. Tyrosinase Inhibitory Activity of Gla-ZIF-8

The tyrosinase inhibitory activities of glabridin and Gla-ZIF-8 were measured using L-DOPA as a substrate [36]. Firstly, glabridin and Gla-ZIF-8 were dissolved separately in ethanol, and then diluted to various concentrations with PBS buffer (0.05 mM, pH = 6.8). Then, a solution of 1 mL L-DOPA (1.0 mM) in PBS and 0.5 mL of the test sample (containing various concentrations of Gla or Gla-ZIF-8) were added to test tubes and continuously agitated for 5 min at 37 °C. Then, 0.5 mL of tyrosinase (100 U/mL) in PBS was added, and the reaction was continuously agitated for 10 min. The absorbance at 475 nm was measured with a UV-2450 spectrophotometer, with a blank reference of 1 mL L-DOPA solution, 0.5 mL of tyrosinase, and 0.5 mL PBS. The tyrosinase inhibition activity was calculated according to Equation (2):

(2) $$\mathrm{Inhibition}\%=\frac{A_{i}}{A_{0}}\times 100\%$$

where A_i_ represents the absorbance of the test sample, while A_0_ represents the absorbance of the control with no added sample at 475 nm.

### 3.7. In Vitro Anti-Oxidant Activity

The in vitro anti-oxidant activities of glabridin and Gla-ZIF-8 were evaluated by conducting a DPPH free radical-scavenging capacity experiment according to the method reported by Jirawattanapong et al., with some modifications [36]. Specifically, 0.5 mL of the test sample solution with various concentrations of glabridin and Gla-ZIF-8 (2.5~80 μg/mL) was added into 1 mL DPPH in ethanol (0.2 mM), and then incubated for 30 min in darkness. The decrease in absorbance at 517 nm was measured with a UV-2450 spectrophotometer, with a blank reference of 1 mL DPPH solution and 0.5 mL ethanol. The scavenging capacity was calculated using Equation (3):

(3) $$\mathrm{Inhibition}\%=\frac{A_{i}}{A_{0}}\times 100\%$$

where A_i_ represents the absorbance of the test sample, while A_0_ represents the absorbance of the control with no added sample at 517 nm.

### 3.8. Cellular Anti-Oxidant Activity

The anti-oxidant activity of glabridin and Gla-ZIF-8 in living cells was evaluated as reported previously [34,35]. For this study, the human gastric cancer cell line MGC-803 was chosen to evaluate the cellular anti-oxidant activity (CAA). After digestion with trypsin, MGC80-3 cells in the logarithmic growth phase were collected and counted, and the cell concentration was adjusted to 4000 cells/mL. For determination of antioxidant activity, 90 μL of cell suspension was seeded into a 96-well plate and incubated for 12 h. Then, 10 μL of fresh DMEM medium containing glabridin, ZIF-8, or Gla-ZIF-8 was added to each well (equivalent concentration of glabridin as 4 μg/mL and the same amount of ZIF-8) and incubated for 24 h. Thereafter, the cells were washed thrice with PBS, the medium was replaced with 100 μL of fresh medium without FBS containing 25 μM DCFH-DA, and the cells were incubated for 1 h. Finally, the wells were washed three times with PBS, and 100 μL of ABAP (600 μM) in PBS was added to each well. Samples were measured with a microplate reader every 5 min. The detection time lasted 1 h and the excitation and emission wavelengths were 485 nm and 535 nm, respectively. The negative controls only contained DCFH-DA and ABAP, while the blank controls only contained DCFH-DA. The resultant time-fluorescence intensity curve was integrated to obtain the area, and then the CAA value was calculated according to Equation (4).

(4) $$\mathrm{CAA}=100-\frac{\left(\int A_{s}-\int A_{b}\right)}{\left(\int A_{c}-\int A_{b}\right)}\times 100\%$$

where ∫A_s_ represents the test sample group; ∫A_c_ represents the experimental control; and ∫A_b_ represents the experimental blank.

### 3.9. Statistical Analysis

All experiments were repeated three times, and the data were analyzed using Origin 8.5 software. The results are presented as mean ± SD. The level of statistical significance for all tests was taken as *p* < 0.05.

## 4. Conclusions

In this study, we proposed a method for encapsulating insoluble drugs with ZIF-8 using the anti-solvent precipitation method. TEM and SEM showed that Gla-ZIF-8 had a regular fusiform or cruciate flower-like structure, with a size of 3 μm. The results of UV, zeta potential, FT-IR, XRD, and TGA analysis suggested that glabridin was successfully encapsulated by ZIF-8. The melanogenesis inhibition activity of glabridin was enhanced by protective encapsulation within ZIF-8. In addition, there was no significant difference in the in vitro anti-oxidant activities of free glabridin and Gla-ZIF-8, while the intracellular anti-oxidant activity of Gla-ZIF-8 was much higher than that of free glabridin. This was because the encapsulation of ZIF-8 enhanced the mutual attraction between cells, then improved the internalization of Gla-ZIF-8 into cells and the bioavailability of glabridin. In addition, the successful preparation of Gla-ZIF-8 confirmed the applicability of drug loading using seeded-grown ZIF-8 to hydrophobic drugs.

## Data Availability

Not applicable.

## Sample Availability

Due to the current epidemic situation, samples are temporarily unavailable.
